# IL-32 and IL-34 in hepatocellular carcinoma

**DOI:** 10.3389/fmed.2022.1051113

**Published:** 2022-11-11

**Authors:** Yang Si, Jiwei Zhang, Shisan Bao, Steven G. Wise, Yuli Wang, Yanfang Zhang, Yuhong Tang

**Affiliations:** ^1^Department of Hematology, Shanghai Ninth People's Hospital, Shanghai Jiaotong University School of Medicine, Shanghai, China; ^2^Department of Cardiothoracic Surgery, Songjiang Hospital Affiliated to Shanghai Jiaotong University School of Medicine (Preparatory Stage), Shanghai, China; ^3^Faculty of Medicine and Health, School of Medical Sciences, The University of Sydney, Sydney, NSW, Australia

**Keywords:** IL-32, IL-34, hepatocellular carcinoma, biomarkers, gastrointestinal

## Abstract

Hepatocellular carcinoma (HCC) remains a major challenge to clinicians due to its unacceptably high mortality and morbidity. The etiology of HCC is multi-faceted, including viral infection, alcoholism and non-alcoholic fatty liver disease. Dysregulated host immunity contributes to tumorigenesis among these susceptible individuals with pre-existing condition(s). IL-32 and IL-34 are key cytokines driving the development of chronic inflammatory conditions such as rheumatoid arthritis, systemic lupus erythematosus, as well as chronic liver diseases. IL-32 and IL-34 play an important role augmenting the development of HCC, due to their direct influence over host inflammation, however, new roles for these cytokines in HCC are emerging. Here we comprehensively review the latest research for IL-32 and IL-34 in HCC, identifying a subset of potential therapeutic targets for use in precision medicine.

## HCC

Hepatocellular carcinoma (HCC), the most common primary liver cancer, continues to have unacceptably high mortality and morbidity (1), as diagnosis at later stages of disease is common and distant metastasis prevalent. The etiology of HCC is complex, with a number of risk factors including chronic hepatitis B and/or C viral infection (2), followed by non-alcoholic liver disease or diabetes mellitus (3). The incidence of new HCC caused by hepatitis viruses decreases gradually with age, due to increasing rates of vaccinations against viral infection and/or application of effective anti-viral medications for infected individuals (2). The precise underlying mechanism of HCC remains to be elucidated, but the causes of HCC likely include genetical susceptibility, viral triggers, alcoholic or non-alcoholic hepatic persistent damage and dysregulated host immunity trigging tumorigenesis, particularly among susceptible individuals with these pre-existing condition(s) (4). Cirrhosis caused by any trigger is a chief driver for the development of HCC (2). Although liver biopsy remains the gold standard for accurate diagnosis of HCC, it is not always acceptable to patients and/or doctors, due to its invasive nature. Alternative non-invasive approaches use a combination of routine biochemistry and sonography with relatively lower sensitivity and specificity (5). Thus, exploring biomarkers with more sensitivity and specificity for the detection of HCC at an early stage would be ideal for the management of such a devastating disease. In addition, hepatocarcinogenesis is very complicated, but host inflammation/immunity in the local affected livers of the susceptible individuals contributes to the pathogenesis of liver damage, repair and mutation, eventually progressed toward malignancy (6).

## IL-32

IL-32 was originally named natural killer cell transcript 4 (NK4) because of its selective production by activated NK cells and T cells (7), is considered to be a pro-inflammatory cytokine, because IL-32 stimulates production of NK cells and other leucocytes as well as amplifying the activity of inflammatory mediators, such as TNF, IL-1β, and IL-6 (8). In *in vitro* assays, IL-32 stimulates NK cells to produce pro-inflammatory cytokines. In addition, IL-32 is substantially co-localized with pro-inflammatory cytokines (TNF, IL-1β, IL-6) in inflamed tissues in the context of inflammatory bowel disease, in synovial tissues from rheumatoid arthritis or serum from psoriasis patients (9). Taken together, this data strongly suggests that IL-32 promotes inflammation in the focal regions of diseased tissues. However, it remains to be clarified if IL-32 is enhancing or suppressing the development of malignant tumors, which will be further discussed below. This is linked with another proinflammatory cytokine, IL-34, for the pathogenesis in HCC, and will be reviewed below.

## IL-34

IL-34, a member of interleukin 1 family, is also classified as a pro-inflammatory cytokine. IL-34 is secreted by a number of cells including macrophages, fibroblasts, and hepatocytes (10). The close structural homology between IL-34 and CSF-1 enables both IL-34 and M-CSF to promote differentiation, proliferation and survival of mononuclear cells *via* CSF-1R (11). Like IL-32, IL-34 is also up-regulated in diseased tissues including for inflammatory bowel disease (12), rheumatoid arthritis (13), and ischemia/reperfusion injury driven acute kidney injury (14), however it is down-regulated in gastric cancer (15). M-CSF contributes to the survival, proliferation and differentiation of mononuclear phagocytes (16). Interestingly, a close correlation is observed between IL-34 and M-CSF in liver injury among chronic hepatitis C patients who had high fibrosis scores and possibly cirrhosis (10). Furthermore, serum IL-34 is correlated with hepatic inflammation and fibrosis in chronic hepatitis B patients (17), which are considered to be major markers of the development of cirrhosis and liver malignancy.

## Host immunity and cancer

It is well-known that host immunity plays a critical role in carcinogenesis (18), however the mechanisms are complex and it is not simply a matter of host immunity protecting or augmenting the development of malignancy. While not yet fully understood, it is likely that host immunity status, whether pro-inflammatory or anti-inflammatory, in the microenvironment during the development of malignancy, either boosts or inhibits the growth of a cancer (19). IL-32 and IL-34 are two relatively new pro-inflammatory cytokines, involved in inflammation/immunity in the host. The interaction among tumor cells, macrophages, activated endothelial cells and secreted cytokines plays an important role in determining tumor development in the microenvironment (20). Cytotoxic T cell mediated pro-inflammatory immunity contributes to inhibition/killing of cancer; whereas regulatory T cells promote the development of cancer (21). Furthermore, the role of tumor associated macrophages (TAMs) in the microenvironment has been the subject of considerable debate (22). TAMs enhance tumor invasion directly, promote formation of new blood vessels and lymphatic vessels, and promote migration of tumor cells indirectly (23), perhaps by suppressing host immunity/inflammation (24). Interestingly, it has also been reported that TAMs inhibit cancer growth and metastasis (25). The differential roles of TAMs likely relate to their terminal differentiation into classically activated M1 macrophages and alternatively activated M2 macrophages based on the surface biomarkers and their functionalities (22). M1 or M2 TAMs are promoting to activity of Tc or Treg cells in response to the different microenvironments (26).

## IL-32 and HCC

There is a constitutive level of intra-hepatic IL-32 expression in the non-cancer liver (27, 28). The expression of intra-hepatic IL-32 is significantly upregulated in the livers of HCC patients at both an mRNA and protein level, particularly near the vascular invasive region (27), suggesting that IL-32 augments local invasion and/or distance metastasis. The elevated intra-hepatic IL-32 is localized in the cytoplasm and nucleus, suggesting IL-32 acts in autocrine and paracrine fashions during the development of HCC. The upregulated intra-hepatic IL-32 is consistent with increased serum IL-32 from HCC patients, compared to that of non-HCC controls (27). Interestingly, serum IL-32 is gradually increased from the cohorts of the control, acute hepatitis, chronic hepatitis, liver cirrhosis, and HCC (27), further linking IL-32 and its pro-inflammatory effects with the severity and duration of hepatic damage. Using siRNA to suppress IL-32, the proliferation and survival of HCC cell lines is inhibited *via* classical p38 MAPK and NF-kB pathways, adding further evidence that IL-32 augments the development of HCC. In addition, suppressing IL-32 contributes to caspase mediated apoptosis of HCC cells, which is in line with the concept that caspases are important in activating target cells to undergo apoptosis (29) and/or pyroptosis (30).

Furthermore, some correlations between serum IL-32 and clinicopathological parameters in HCC patients have been observed (28). A relationship between circulating IL-32 and metastasis or local invasion (28) has been reported, suggesting that IL-32 is critical in local invasion or distance metastasis perhaps *via* inhibiting NK or TCL activities against HCC (31). Another research team reports a correlation between circulating IL-32 and the area of VEGF staining in the HCC tissues, suggesting that IL-32 may also augment the development of HCC *via* boosting neovascularisation, which offers some evidence for the management of HCC patients with combined anti-IL32 and anti-VEGF therapy (32).

Surprisingly, no correlation has yet been detected between IL-32 and age or sex, though only one limited study has so far been reported, with included patients of a maximum of 60 years old, with the number of HCC patients from the younger group being almost 4 times more than these from the older cohort (72 vs. 28) (28). Moreover, the poor gender balance of this study (male 80 vs. female 20), was a further limitation. In addition, most of the study patients were >50 years old, who likely have almost no benefit from estrogen in reducing the incidence of malignancy, a phenomenon seen in females of fertile age (33, 34). The small sample size also contributed the absence of significant statistical difference among patients stratified into well, moderate and poor differentiations (*n* = 16, 60, 24). Similarly, the small sample size was also likely a driver of a lack of significant difference detected between single and multiple HCC tumors, or alpha-fetoprotein (AFP) higher vs. low, or the different causative agents (HBV, HCV, or others) (28). Thus, it remains desirable to more thoroughly investigate the role of IL-32 in the oncogenesis of HCC in future studies by extending the sample size of the HCC patients, including healthy cohorts from multiple centers and as well as patients from different racial backgrounds (28).

Similarly, there was no significant difference in IL-32 levels between HCC patients with and without cirrhosis, although the sample number was relatively large (60 vs. 40) (28). This is supported by a report showing that disturbed local immunity augments the tumorigenesis and development of HCC (2), independent of the presence or absence of cirrhosis. Further, IL-32 has an established role in the development of non-alcoholic fatty liver disease (35) and in attenuating experimental induced liver injury (36, 37), providing additional evidence for the pro-inflammatory role of IL-32 in targeted tissue (liver), possibly initiating tumorigenesis in this context, among genetically susceptible individuals. There is currently no information available about the correlation between IL-32 expression and grade/stage of HCC or tumor size, which should be explored in future. Taken together, IL-32 is a promising target for the development of novel therapeutic targets for precision medicine in the management of inflammatory-driven liver disease including HCC.

## IL-34 and HCC

There is a constitutive level of intra-hepatic IL-34 expression in healthy hepatocytes (5). The expression of intra-hepatic IL-34 is significantly upregulated in the livers of HCC patients (5), localized in the cytoplasm of hepatocytes, compared to that of non-cancer cohorts (5). This is further supported by evidence of a correlation between elevated intra-hepatic IL-34 and serum IL-34 from HCC patients, compared to non-HCC cohorts (5). Based on these studies, it is suggested that IL-34 acts in both autocrine and paracrine fashions (5) to augment the development of HCC. This notion is supported by observations of significantly higher intra-hepatic IL-34 detected in HBV induced HCC patients, compared non-cancer or chronic hepatitis B or hepatitis B viral induced-cirrhosis patients, particularly that IL-34 is localized in the cytoplasm of hepatocytes of HCC (5). Interestingly, intra-hepatic IL-34 is lower in the liver of chronic hepatitis B or cirrhosis patients, compared to healthy cohorts (5), suggesting that the pathophysiological function of the hepatocytes from the inflamed liver (chronic hepatitis B liver) or cirrhotic liver is compromised leading them to produce less IL-34. AFP is a biomarker which has been routinely applied in the clinic for diagnosis of HCC, although it is challenged for its accuracy and sensitivity (38). Significant differences in intra-hepatic IL-34 between normal and higher AFP HCC patients, further support that IL-34 promotes the development of HCC (5).

Strong correlations between IL-34 and clinicopathology from HCC patients provides further evidence of a role for IL-34 in the development of HCC. There is a positive correlation between intra-hepatic IL-34 and the size of HCC, when the tumor size is smaller than 5 cm (5), but an inverse correlation with HCC if the tumor size is >5 cm. This could be due to intra-hepatic IL-34 being partially derived from hepatocytes from HCC patients if the HCC tumor size is smaller than 5 cm, a scale that would allow for sufficient blood supply and nutrition, maintaining pathophysiological conditions, and allowing IL-34 to act as an autocrine and paracrine factor. However, the microenvironment would be disrupted when the HCC tumor size is >5 cm due to nutritional competition and/or physical compression due to space limitations (39), resulting in central necrosis of the large HCC.

This is supported by the finding that circulating IL-34 is significantly reduced in the HCC patients following transarterial chemoembolisation (TACE), which is minimally invasive but may cause the destruction of substantial numbers of HCC cells in the affected liver following the local intervention (40). It is common that HCC patients treated with TACE often have multiple, large sized HCC tumors and may not be suitable for surgical resection. However, there is no significant downregulation of circulating IL-34 from the HCC patients prior to and post-surgery (5). This may be due to the factor that the HCC patients who were eligible for surgery had relatively small in size of HCC or a smaller number of tumors. Thus, surgical resection of HCC tumor may not have major impact on IL-34 production, compared to the HCC patients had TACE (5). Recent advances and development in surgery, may change the surgical indications and/or suitability for HCC patients in the future. Furthermore, circulating IL-34 seems to be a reliable biomarker for prognosis of non-viral related HCC patients (41), but not HBV induced HCC (5). Such findings suggest there is differential role of IL-34 during the development of HCC among patients with different etiologies. Since IL-34 seems to act in autocrine and paracrine fashions during the development of HCC, targeting IL-34 either systemically or *via* TACE may offer a therapeutic approach for the management of HCC.

Although the information above suggests that IL-32 and IL-34 promote the development of HCC, it remains to be explored whether there is alteration of local and/or circulating IL-32 or IL-34 following chemo- and/or radiotherapy. However, we speculate that both local and/or circulating IL-34 would decrease following chemo- and/or radio- therapy, which is supported by the finding that IL-34 is significantly decreased after transarterial chemoembolisation (40). Such information would be useful to understand the effective of the treatment(s) and monitor disease progression.

Searching bioinformatics databases to investigate the underlying mechanism of IL-34 during the development of HCC, identifies that miR-28-5p is targeting IL-34 (42), which is consistent with the finding of an inverse correlation between miR-28-5p and metastasis, recurrence, and poor survival. To confirm the role of IL-34 during the development of HCC, miR-28-5p transfected HCC cells which interfere with the function of downstream IL-34 were studies (42). In a xerograph model, tumors were significantly increased in the recipients (nude mice) of miR-28-5p transfected HCC cells, compared to that of mock-transfected HCC cells, accompanied with reduced TAMs (42). This finding provides further evidence that IL-34 augments the development of HCC *in vivo*. However, there is room for improvement from the study of Zhou et al. in that the recipients are nude mice, which are lacking host immunity and unable to mount an effective host defense, despite the positive outcomes in manipulating miR-28-5p related IL-34 in the development human HCC (42). Thus, this early finding should be further verified in humanized animals with full immunity in the future, particularly inoculating *via* the portal vein to better mimic real-world conditions, to investigate the role of the hosts local and systemic immunity in the augmentation of HCC, particularly in relationship with IL-34, perhaps using IL-34 manipulated animals.

A correlation between intra-hepatic IL-34 and load of HBV from HBV induced HCC patients has also been observed, suggesting local and systemic IL-34 participates in the host immunity against HBV infection and subsequent inflammation, and eventually toward malignancy following long-term persistent liver damage (5). It is well-known that HBV is a major cause of HCC in Asia, particularly in China; whereas HCV is a more common contributing factor for the development of HCC in Western countries. Intra-hepatic IL-34 is substantially increased in HCV induced fibrosis compared to healthy cohorts (10), perhaps *via* enhancing hepatic satellite cells to produce collagen in the microenvironment. HCV induced inflammation augments hepatocytes to produce IL-34 and macrophage colony-stimulating factor (M-CSF) (10); whereas M-CSF is important in recruitment of macrophages (Kupffer cells in the liver). Another important point is that there is huge amount of recruitment of macrophages in the liver from the HCV liver *via* production of MCSF (10).

There is also a correlation between intra-hepatic TAMs and HCC differentiation or the number of HCC tumors in the liver (5), suggesting that intra-hepatic TAMs from the HCC provide protection during the development of HCC. Interestingly there is no significant difference in total TAMs between healthy control and HBV induced HCC (5), although there is significantly lower Kupffer cells from CHB and cirrhotic patients. Reduced Kupffer cells from CHB or cirrhotic patients, compared to the healthy control, perhaps is due to compromised local immunity from CHB (43) or cirrhotic patients (44). The explanation for no significant difference of Kupffer cells between the healthy control and HCC (5) is such: although it isn't sufficiently effective, local immunity is being boosted for killing of malignant hepatocytes during the development of HBV induced HCC, resulting in recruitment of circulating macrophages into the microenvironment. In addition, as stated above, it is unknown which subset of these Kupffer cells (TAMs) dominate in the liver from HCC patients. TAMs are classified as M1 and M2 with distinctive roles during the development of cancer. However, there is no classification of these TAMs from the study (5), which should be explored in future and may offer the potential role of these TAMs and the correlation with survival.

However, observations about the role of IL-34 in other cancer types has so far shown contrary trends. For instance, in gastric cancer IL-34 expression is significantly reduced, compared to the paired non-cancer tissues (45), although there is no significant difference in circulating IL-34 between gastric cancer patients and healthy control. This observation in the context of gastric cancer suggests that IL-34 plays a protective role during the development of malignancy, in contrast to that in HCC. The discrepancy of IL-34 expression between HCC and gastric cancer may be related to the fact that these are different organ systems with almost completely different microenvironments (46), despite both liver and stomach being covered with epithelial cells and belonging to the digestive system. Since IL-34 is an independent biomarker for predicting the development of gastric cancer (15), as well as an important regulating factor for the differentiation of HCC (45), IL-34 may be a therapeutic target in the management of HCC or gastric cancer, but in the almost completely opposite way, i.e., boosting or inhibiting the expression of IL-34, in application of targeting of IL-34 for precision medicine.

## Conclusion

**Figure 1:**
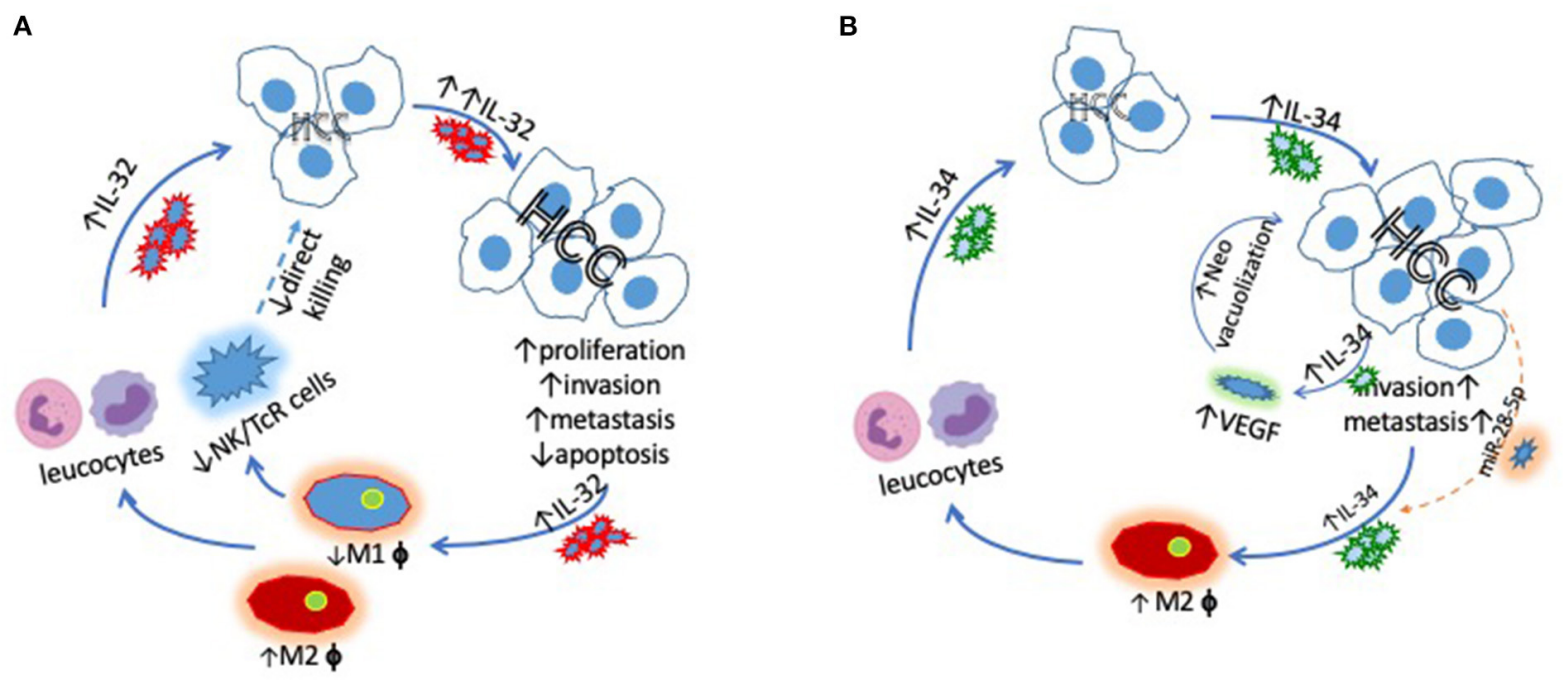
Schematic demonstrating **(A)** the key interactions between IL-32 and HCC driving increased HCC proliferation, invasion and metastasis *via* promoting M2, but inhibiting M1 cells and **(B)** the links between IL-34 and HCC, which also ultimately lead to increased HCC tumorigenesis. miR-28-5p blocks the pro-tumor effects of IL-34.

We conclude that IL-32 contributes to the tumorigenesis of HCC in both autocrine and paracrine fashions by promoting local invasion and/or distance metastasis, confirming evidence that siRNA suppressed IL-32 inhibits HCC cell proliferation *via* caspase mediated apoptosis. The effectiveness of anti- IL-32/VEGF targeted therapy is justified by close correlation between intra-hepatic IL-32 and VEGF from HCC patients. Furthermore, the inverse correlation between IL-34 and 5-year survival implies that IL-34 augments HCC development, particularly among HBV induced HCC in paracrine and autocrine fashions. There is a differential role for IL-34 during the development of HCC among patients with different etiologies. Targeting IL-34 systemically or *via* TACE may offer a novel therapeutic approach for the management of HCC. The role of TAMs in HCC remains to be explored, depending on the subset of TAMs, i.e., M1 for inhibiting but M2 for promoting the development of HCC. A schematic figure has been added to illustrate the interaction among these factors (Figure 1). The precise relationship among IL-32, IL-34, and TAMs will be determined in the future with important implications for precision medicine.

## Author contributions

YS and JZ wrote the manuscript. SB and SW revised the manuscript. YW, YZ, and YT concept the manuscript. All authors contributed to the article and approved the submitted version.

## Conflict of interest

The authors declare that the research was conducted in the absence of any commercial or financial relationships that could be construed as a potential conflict of interest.

## Publisher's note

All claims expressed in this article are solely those of the authors and do not necessarily represent those of their affiliated organizations, or those of the publisher, the editors and the reviewers. Any product that may be evaluated in this article, or claim that may be made by its manufacturer, is not guaranteed or endorsed by the publisher.
